# *Bifidobacteria* Abundance-Featured Gut Microbiota Compositional Change in Patients with Behcet’s Disease

**DOI:** 10.1371/journal.pone.0153746

**Published:** 2016-04-22

**Authors:** Jun Shimizu, Takao Kubota, Erika Takada, Kenji Takai, Naruyoshi Fujiwara, Nagisa Arimitsu, Yuji Ueda, Sueshige Wakisaka, Tomoko Suzuki, Noboru Suzuki

**Affiliations:** 1 Department of Immunology and Medicine, St. Marianna University School of Medicine, Kawasaki, Japan; 2 Department of Medicine, the Japan Self Defense Forces Central Hospital, Tokyo, Japan; National Cancer Institute, UNITED STATES

## Abstract

Gut microbiota compositional alteration may have an association with immune dysfunction in patients with Behcet’s disease (BD). We conducted a fecal metagenomic analysis of BD patients. We analyzed fecal microbiota obtained from 12 patients with BD and 12 normal individuals by sequencing of 16S ribosomal RNA gene. We compared the relative abundance of bacterial taxa. Direct comparison of the relative abundance of bacterial taxa demonstrated that the genera *Bifidobacterium* and *Eggerthella* increased significantly and the genera *Megamonas* and *Prevotella* decreased significantly in BD patients compared with normal individuals. A linear discriminant analysis of bacterial taxa showed that the phylum *Actinobacteria*, including *Bifidobacterium*, and the family *Lactobacillaceae* exhibited larger positive effect sizes than other bacteria in patients with BD. The phylum *Firmicutes* and the class *Clostridia* had large effect sizes in normal individuals. There was no significant difference in annotated species numbers (as numbers of operational taxonomic unit; OTU) and bacterial diversity of each sample (alpha diversity) between BD patients and normal individuals. We next assigned each sample to a position using three axes by principal coordinates analysis of the OTU table. The two groups had a significant distance as beta diversity in the 3-axis space. Fecal sIgA concentrations increased significantly in BD patients but did not correlate with any bacterial taxonomic abundance. These data suggest that the compositional changes of gut microbes may be one type of dysbiosis (unfavorable microbiota alteration) in patients with BD. The dysbiosis may have an association with the pathophysiology of BD.

## Introduction

Behcet’s disease (BD) is a systemic inflammatory disease, characterized by recurrent attacks of oral aphthosis, genital ulcers, skin lesions and uveitis. In the BD lesions, neutrophilic and lymphocytic infiltrations emerge.

The etiology of BD is largely unknown and dysregulation of immune system is thought to associate with development and maintenance of BD [1].

It is well known that HLA-B51 is associated with BD [2]. Recent genome wide association studies (GWAS) suggested several cytokine genes and their receptor genes as disease susceptibility genes [3, 4].

Heat shock protein (HSP) functions as an intracellular chaperonin of other proteins and significant sequence homology is found between mammalian HSP and microbial HSP [5]. HSP was thought to be a major cause of the skewed immune responses in patients with BD because of the molecular mimicry between human HSP and microbial HSP [5].

We found that colon tissues of BD patients expressed HSP excessively where mononuclear cells infiltrated [6]. T cells of BD patients located near the infiltrating cells and they responded to specific epitopes of HSP [7].

Recently, we have reported that T helper 17 (Th17) cells increased and had already been activated in vivo in patients with BD [8, 9]. Th17 cells were suggested to be highly pathogenic T cells in human autoimmune diseases and BD [10–14].

The colonization of several bacteria in the intestine activated Th17 cells of experimental models of infection [15] and autoimmune diseases [16, 17].

Metagenomics is a field of research where genomic DNA obtained from bacteria are analyzed. The whole bacterial genome is termed as microbiome and includes anaerobes which are hardly cultivated in the clinical laboratories [18]. Next generation sequencing device with metagenomic analysis makes it possible to characterize individual bacterial genomes obtained from clinical samples [19].

Analyses of the gut microbiomes are important for stably assessing intestinal environment of human diseases [20, 21]. We utilized the technique to estimate whether altered gut microbiota composition existed in patients with BD.

## Materials and Methods

### Patients

We studied 12 patients (7 women and 5 men) with BD. Their mean ± SD age was 48.8 ± 17.5 years (range, 18–78 years). We followed the patients for 9.3 ± 5.1 years (range, 1–16 years) from the time of disease onset. Patients fulfilled the diagnostic criteria proposed by the International Study Group of BD [22]. Table 1 summarizes the clinical characteristics and medications of the patients at the time of sample collection and throughout the entire disease courses.

**Table 1:** Demographical and clinical characteristics of patients with Behcet’s disease and normal individuals.

Characteristics at the time of sample collection	Behcet’s disease (BD, n = 12)		Normal individuals (NI, n = 12)
Age, mean years (range)	48.8 (18–78)		48.6 (25–76)
Men: Women	5:7		6:6
	At the time of sample collection	During the entire disease course	At the time of sample collection
**Disease activity parameters**			
CRP, mean ± SD, mg/dL	0.28 ± 0.56	NA	NA
Oral aphthosis, %	100	100	0
Skin involvement, %	92	100	0
Genital ulcers, %	50	83	0
Uveitis, %	42	92	0
Gastrointestinal system involvement, %	17	25	0
Central nervous system involvement, %	17	33	0
Vascular involvement, %	17	17	0
Arthritis, %	8	100	0
**Medications**			
Colchicine, %	100	100	0
Steroid, %	42	50	0
Cyclosporine, %	17	25	0
Azathioprine, %	8	8	0
Cyclophosphamide, %	0	8	0
Methotrexate, %	0	0	0
Biologic agents, %	0	0	0

NA, not applicable and/or not available.

All patients had oral aphthosis and 11 of 12 patients had skin involvement at the time of sample collection. In this study, the two patients were fecal occult blood testing positive but none showed obvious symptoms of the gastrointestinal tract. Behcet’s Disease Activity Index (BDAI) [23] was 6.9 ± 2.3 at the time of sample collection. We excluded patients treated with intermediate—high dose corticosteroid therapy to minimize the effects on the intestinal environment to the similar extent with intrinsic glucocorticoid [24]. Low dose colchicine (daily doses, 0.5–1.0 mg) seemed not to have a significant effect on the intestinal flora [25–27]. Daily steroid doses were 5.0 mg or less (3.8 ± 1.3) and daily colchicine doses were 1.0 mg or less (0.79 ± 0.26). We treated 6 patients (50%) with a cumulative steroid dose of 143 ± 94.6 g (range, 2.0–270 g) for 8.2 ± 5.1 years (range, 2.5–15 years).

Age and sex matched 12 normal individuals (NI) donated feces and served as control subjects. Exclusion criteria applied to the two groups were as follows: recent (<6 months prior to the sample collection) treatment with probiotics and antibiotics, history of malignancies, intra-abdominal surgical interventions and metabolic disorders such as gout, obesity and diabetes. Individuals involved in this study ate Japanese conventional foods and high meat eaters and vegetarians were not included in the current study.

This study was approved by the institutional review boards of St. Marianna University School of Medicine and was registered with the University Hospital Medical Information Network-Clinical Trials Registry (UMIN000018937). Our research has been conducted according to the principles expressed in the Declaration of Helsinki. Written informed consent was obtained from each individual prior to enrolment in the study. A copy of the written consent is available for review upon request.

### Sample collection

We obtained a fecal sample (1.0 g) from each individual just after production. Several patients produced the samples at their favorite places and kept them at 4°C until attending a hospital within 12 hours after production.

Each sample was suspended in 20% glycerol (Wako Pure Chemical Industries, Tokyo, Japan)/ PBS and was frozen in liquid nitrogen. We stored the samples at –80°C until use.

### DNA extraction

We extracted and purified DNA from the samples according to a literature with minor modifications [28].

In brief, after thawing, we filtered the samples using 100 μm mesh and washed them with PBS. The bacterial pellets were treated with lysozyme (Sigma-Aldrich Japan, Tokyo, Japan). Then the samples were treated with achromopeptidase (Wako Pure Chemical Industries).

The DNA was purified by SDS (Wako Pure Chemical Industries)/ proteinase K (Merck Japan, Tokyo, Japan) treatment, followed by phenol/chloroform extraction. After incubation with RNase A (Wako Pure Chemical Industries), sample DNA was precipitated with polyethylene glycol solution (Wako Pure Chemical Industries).

The samples were assessed by measuring the ratio of optical density at 260 nm to that at 280 nm (typically 1.66 to 2.1). We then confirmed the amplicon libraries using agarose gel electrophoresis.

### 16S ribosomal RNA (rRNA) gene amplification

We amplified the V1–V2 16S rRNA gene region by primers, namely 27Fmod (5’-CCATCTCATCCCTGCGTGTCTCCGACTCAGNNNNNNNNNNNNNAGRGTTTGATYMTGGCTCAG, containing sequencing adaptor and barcode sequences indicated by N) and 338R (5’-CCTCTCTATGGGCAGTCGGTGA TGCTGCCTCCCGTAGGAGT) with 40 ng of template DNA, according to a procedure reported previously [29].

### Purification, quantification and sequencing of the libraries

We purified the libraries with AMPure XP magnetic purification beads (Beckman Coulter Japan, Tokyo, Japan) and quantified with Agilent 2100 Bioanalyzer (Agilent Technologies Japan, Tokyo, Japan), according to the manufacturers’ recommendations.

We sequenced the amplicon libraries (10 pM) using Ion Torrent PGM (Life Technologies Japan, Tokyo, Japan) and sequencing data were converted to FASTQ files.

### Sequence analysis

We processed the files using QIIME software (version 1.9.1) with the default settings according to a tutorial for the microbiome study [30].

The sequencing data contained approximately 8.4 million reads in total and the average quality read per sample was 285,752. We filtered the reads and subjected to operational taxonomic units (OTU; species grouping according to the sequence) with the cut off similarity of 97%.

We summarized the OTU data into several OTU tables using QIIME software for the evaluation of relative abundance of bacterial taxa [31].

We then estimated microbial alpha and beta diversity using QIIME software [31]. Alpha diversity is defined as the diversity within a community and is mainly measured by Chao 1 and Shannon diversity indexes [31].

Chao1 index estimates richness in species numbers and Shannon index estimates evenness (equitability) of species frequencies [31, 32].

Beta diversity is defined as the distance between communities and we estimated the distance by principal coordinates analysis (PCoA) [30–32]. The analysis assigns each sample to a position in a three dimensional structure to reduce the multiple dimensions of otu_table.biom files using linear conversion formulas. We visualized each PCoA plot in the three dimensional structure and summed up the distances between the pair of plots. We compared the total distances within and between BD patients and normal individuals using a two-sided Student's two-sample t-test as an exploratory analysis of beta diversity [31].

We utilized a file (seqs.fna) filtered by QIIME software for a linear discriminant analysis (LDA) of LEfSe (LDA effect size, explained below). We created an OTU table by pick_closed_reference_otus.py with a Greengenes-formatted database (gg_13_5). We modified the file by dividing each OTU by known/predicted 16S rRNA gene copy number abundance using PICRUSt software (version 1.0.0, normalize_by_copy_number.py), and uploaded it to http://huttenhower.sph.harvard.edu/galaxy [33].

### Secretory IgA (sIgA) ELISA

We centrifuged, decanted and collected fecal supernatants and then stored them at –80°C until use [34]. We measured sIgA concentrations of fecal supernatants (BD = 12, and normal individuals = 9) using an ELISA kit (Eagle Biosciences, Nashua, NH) according to the manufacturers’ protocols.

### Statistical analyses

Each value was expressed as mean ± SD and a P value less than 0.05 was considered significant. We directly compared the relative abundance (expressed as parts per unit) of each taxon and the microbial diversities by using Wilcoxon rank sum test with JMP statistical software 8.0.2 (SAS, Cary, NC).

We followed the calculation with Tukey’s honestly significant difference (HSD) test [35]. We considered that bacterial taxa increased or decreased significantly in patients with BD as compared with those in normal individuals by fulfilling the following criteria simultaneously;

#1: bacterial taxa showing significant differences by the Wilcoxon rank sum test, and

#2: bacterial taxa showing positive Tukey’s HSD values.

We then divided the obtained bacterial taxa into two groups, namely, abundant taxa in BD patients and those in normal individuals using the LEfSe (linear discriminant analysis (LDA) effect size) analytic method [36].

LEfSe is obtained from an algorithm to find significant differences in taxonomic abundance between patients and normal individuals, using a nonparametric Kruskal-Wallis test.

Subsequently, LDA is applied to the significantly abundant taxa and the effect sizes of the taxa are provided by the analyses.

Those taxa bestowed higher log LDA scores than 2.0 are chosen for subsequent plotting. Consequently, a larger LEfSe indicates that the taxon discriminates much better between BD patients and normal individuals.

We compared age and gender in Table 1 with Wilcoxon rank sum test or Fisher’s extract test. Spearman’s rank correlation coefficients were used to assess correlations among bacterial taxonomic abundance, BDAI, serum CRP, daily administered and cumulative steroid doses and sIgA concentrations of fecal supernatants using the JMP software. We corrected the Spearman’s rank correlation coefficients by controlling the Benjamini-Hochberg false discovery rate (FDR, q-value of 0.05) [37].

## Results

### Higher relative abundance of the phylum *Actinobacteria* in patients with BD

We directly compared relative abundance (expressed as parts per unit) of bacterial taxa between patients with BD and normal individuals.

**Fig 1:**
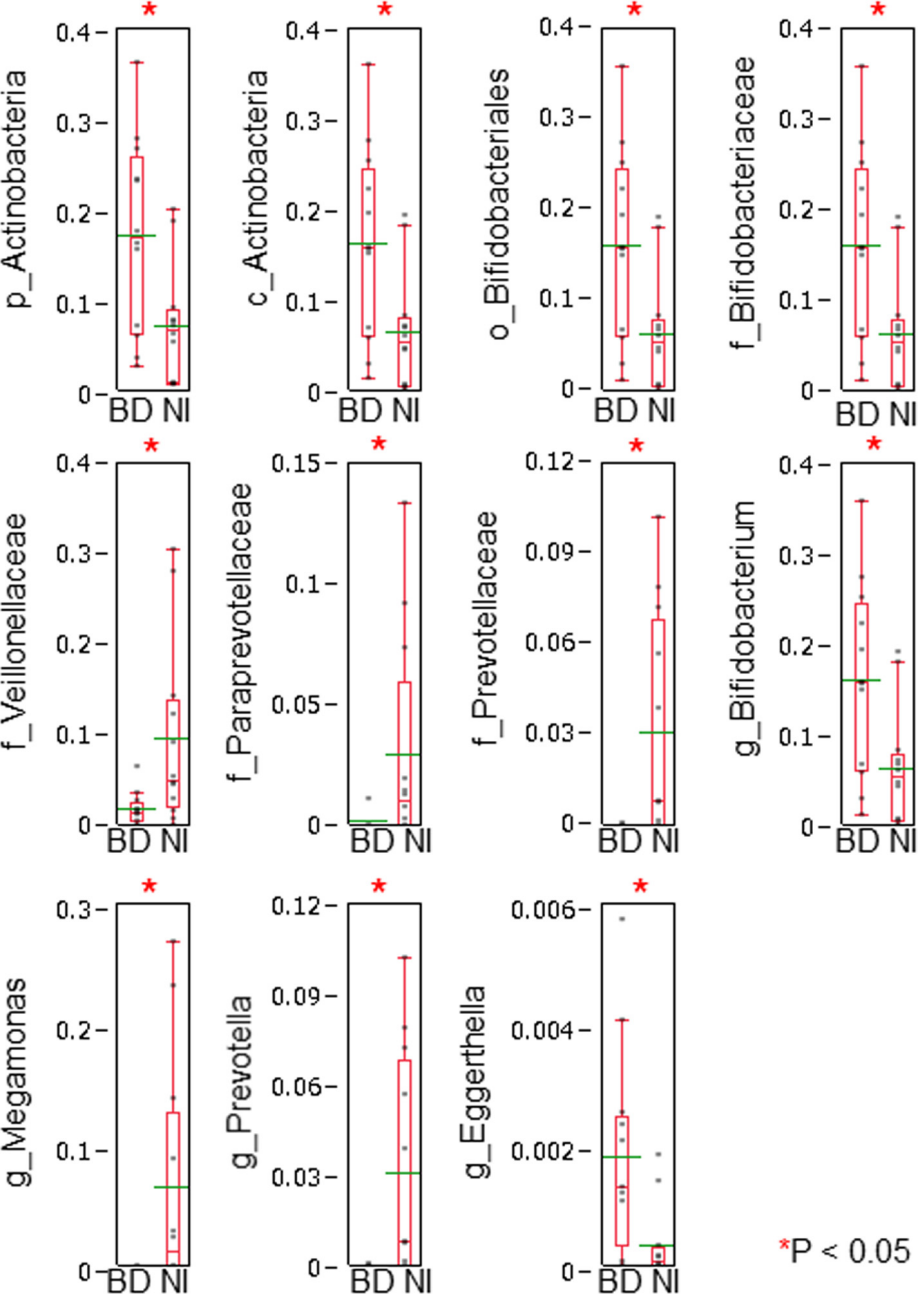
Direct comparison of bacterial taxonomic abundance between BD patients and normal individuals. We compared directly the relative abundance (expressed as parts per unit) between BD patients (BD) and normal individuals (NI). We conducted Wilcoxon rank sum test for the differences in every taxon, followed by Tukey’s honestly significant difference (HSD) test. We considered that bacterial taxa increased or decreased significantly in patients with BD as compared with those in normal individuals by fulfilling the following criteria simultaneously; #1: bacterial taxa showing significant differences by the Wilcoxon rank sum test, and #2: bacterial taxa showing positive Tukey’s HSD values. We found that there were significant differences in 11 bacterial taxa (also shown in Fig 2 with asterisks). “p__”, “c__”, “o__”, “f__” and “g__” indicate phylum, class, order, family and genus, respectively. Relative abundance of bacterial taxa in BD patients and normal individuals were displayed with dot plots. A box-plot and a mean level (green line) of each group of BD patients and normal individuals were indicated.

**Fig 2:**
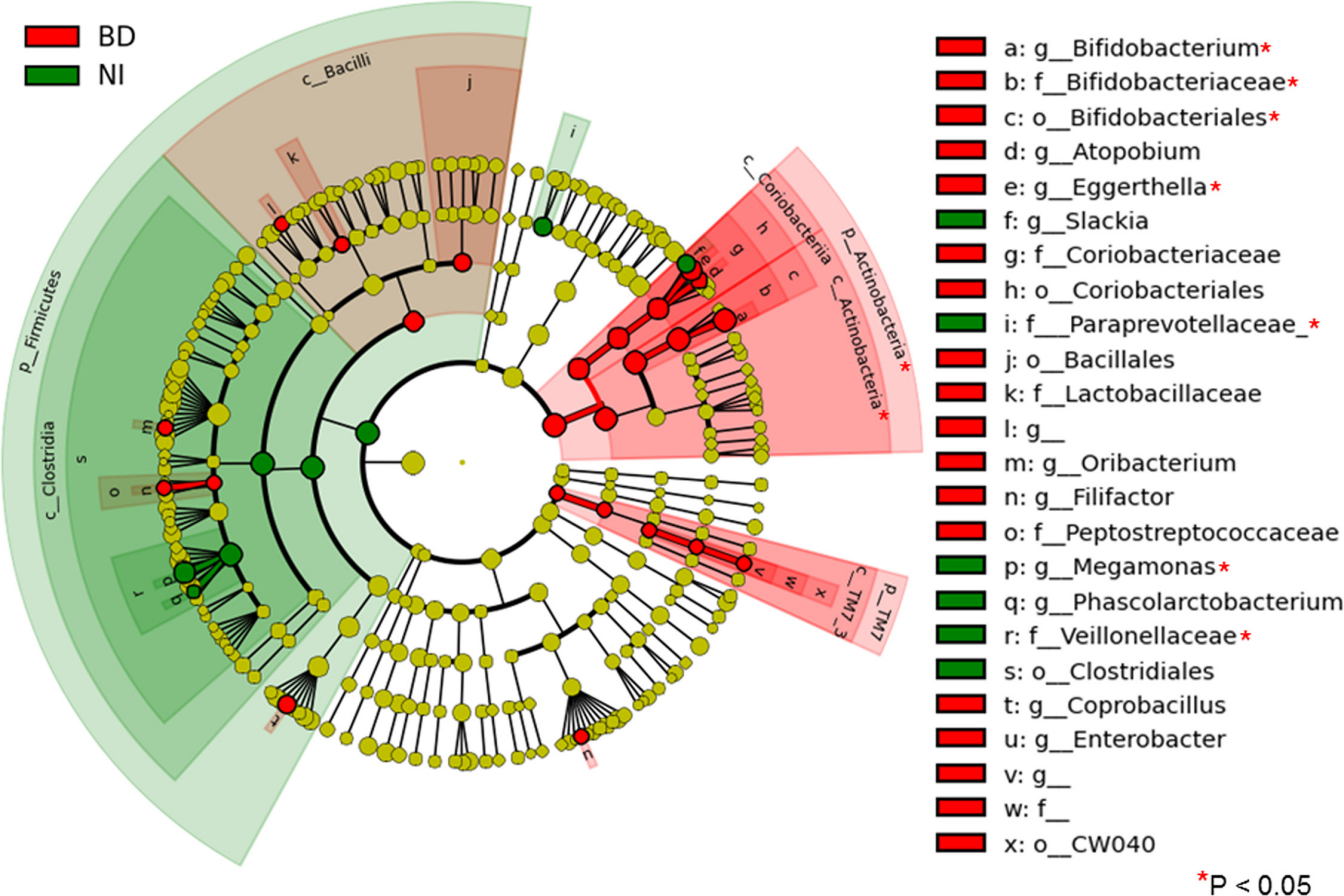
Comparison of the taxa showing different abundance values in BD patients and normal individuals. We analyzed the metagenomic data of bacterial taxa using LEfSe to detect major differences between BD patients (BD) and normal individuals (NI). The LEfSe provides us with cladograms of six-level (from kingdom to genus). Significantly enriched bacterial taxa in samples obtained from BD patients were demonstrated using red circles and shadings. Significantly enriched bacterial taxa in samples obtained from normal individuals were demonstrated using green circles and shadings. In patients with BD, the phylum *Actinobacteria*, namely the classes *Actinobacteria* and *Coriobacteria*, the orders *Bifidobacteriales* and *Coriobacteriales*, and the genera *Bifidobacterium*, *Eggerthella* and *Atopobium* had large effect sizes. The phylum *Firmicutes*, the class *Clostridia*, the order *Clostridiales*, the family *Veillonellaceae*, and the genera *Megamonas* and *Phascolarctobacterium* had large effect sizes in normal individuals.

We conducted Wilcoxon rank sum test and Tukey’s HSD test for the comparison. We found that there were significant differences in 11 bacterial taxa between BD patients and normal individuals (Fig 1 and asterisks in Fig 2).

At the genus level, *Bifidobacterium* and *Eggerthella* increased significantly and *Megamonas* and *Prevotella* decreased significantly in BD patients compared with normal individuals.

We then analyzed the metagenomic data of bacterial taxa using LEfSe analytic method to detect major differences between BD patients and normal individuals. The method provides us with cladograms of six-level (from kingdom to genus, Fig 2).

In BD patients, the phylum *Actinobacteria*, namely the classes *Actinobacteria* and *Coriobacteria*, the orders *Bifidobacteriales* and *Coriobacteriales*, and the genera *Bifidobacterium*, *Eggerthella* and *Atopobium* showed large effect sizes.

Subsequently, the class *Bacilli* and *Lactobacillaceae*, a family of lactic acid bacteria, had relatively large effect sizes in BD patients.

The order *CW040* (of the phylum *TM7*, one of the oral commensal bacteria) also had a large effect size in patients with BD.

The phylum *Firmicutes*, the class *Clostridia*, the order *Clostridiales*, the family *Veillonellaceae*, and the genera *Megamonas* and *Phascolarctobacterium* showed large effect sizes in normal individuals. Subsequently, the family *Paraprevotellaceae* had a large effect size in normal individuals.

### BD prevalent bacterial taxa did not correlate with any BD disease activity parameters

We assessed the relationships among bacterial taxonomic abundance of gut microbiota and several clinical parameters, namely BDAI and serum CRP at the time of sample collection and daily administered and cumulative steroid doses in BD patients. These clinical parameters did not correlate with any relative bacterial abundance in BD patients.

### Increased fecal sIgA concentrations of BD patients did not correlate with any bacterial taxonomic abundance

We measured sIgA concentrations of fecal supernatants to estimate the underlying immunological condition of the intestine in patients with BD by ELISA [35].

**Fig 3:**
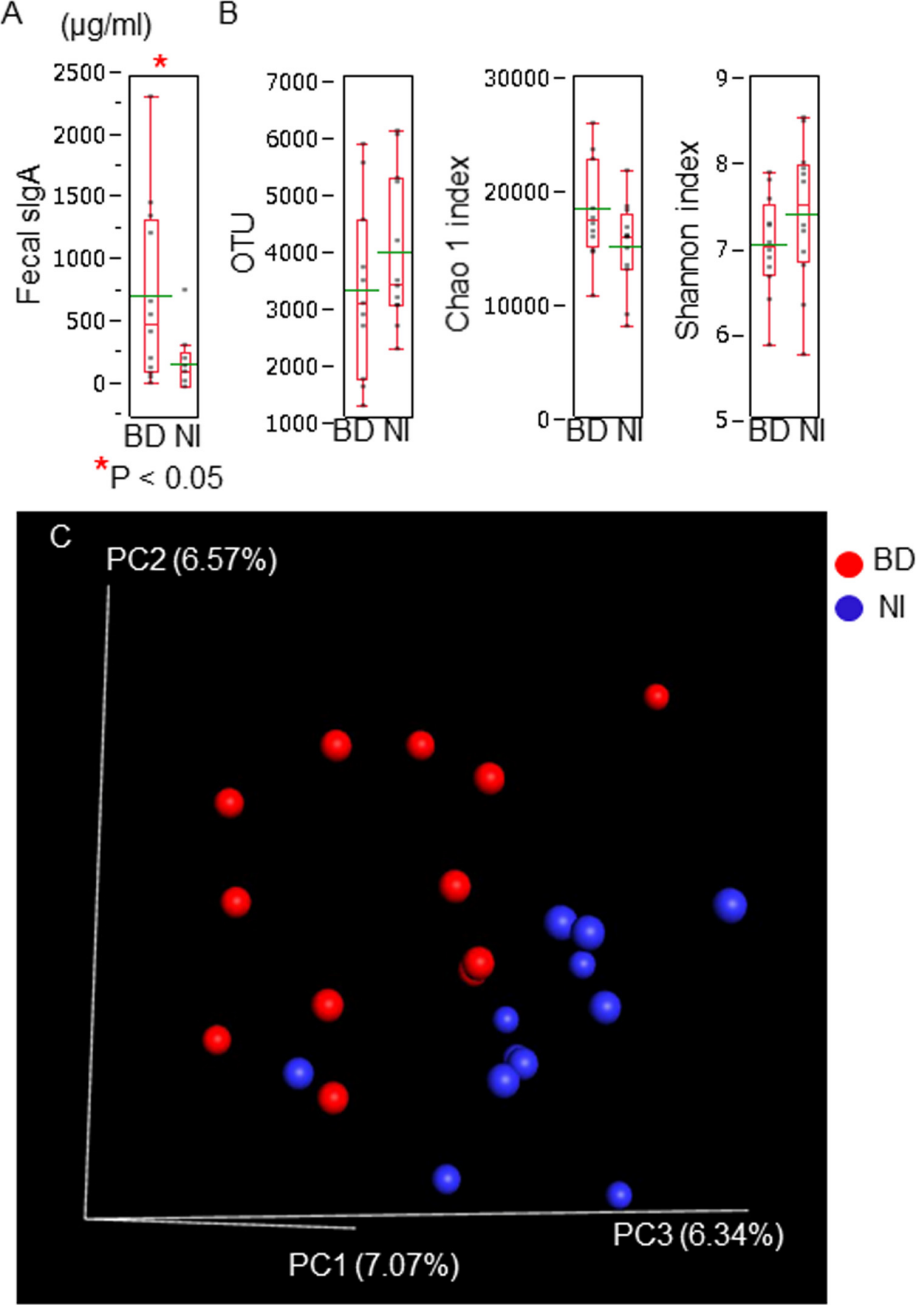
Comparison of fecal secretory IgA concentrations and bacterial diversity between BD patients and normal individuals. (A) We evaluated the secretory IgA (sIgA) concentrations of fecal supernatants using ELISA. We observed a significant increase in sIgA concentrations of patients with BD (BD) compared with those of normal individuals (NI). (B) We counted OTU numbers (annotated species numbers) and estimated alpha diversity score (Chao 1 and Shannon indexes) of each sample. We compared the titers between BD patients and normal individuals. We did not find significant differences in the parameters between BD patients and normal individuals. These biological parameters of BD patients and normal individuals were displayed with dot plots. A box-plot and a mean level (green line) of each group of BD patients and normal individuals were indicated. (C) We estimated beta diversity between BD patients and normal individuals using PCoA of QIIME software with linear conversion formulas. We visualized the PCoA plots in a three dimensional structure where three axes and each contribution ratio (principal coordinate, PC1–3, %) were depicted. We calculated the distance between the distribution of BD patients and that of normal individuals using a two-sided Student's two-sample t-test as an exploratory analysis. We obtained a significant P value of the test of beta diversity between BD patients and normal individuals.

sIgA increased significantly in fecal supernatants of patients with BD compared with that of normal individuals (Fig 3A). We compared the bacterial taxonomic abundance of the OTU tables with sIgA concentrations using the Spearman’s rank correlation coefficients. sIgA concentrations did not correlate with any of bacterial taxonomic abundance in BD patients and normal individuals.

### Analyses of alpha diversity and beta diversity in patients with BD

We counted OTU numbers (annotated species numbers) and estimated bacterial alpha diversity (diversity within an individual) using Chao 1 (richness in species numbers) and Shannon (evenness of species frequencies) indexes of each sample.

The three metagenomic parameters were suggested to be involved in human disease progression [35].

We did not find any significant differences in the OTU numbers and the index scores between BD patients and normal individuals (Fig 3B). The data suggested that bacterial compositional changes of BD patients were not accompanied by the bacterial number and diversity restrictions in this study.

Beta diversity is defined as the distance between communities. We thus estimated beta diversity between BD patients and normal individuals using PCoA, which assigned each sample to a position in a three dimensional structure to reduce the multiple dimensions of the OUT table using linear conversion formulas. Accordingly, beta diversity represents average intergroup distance.

Fig 3C demonstrates the PCoA plots with three axes and each contribution ratio (principal coordinate, PC1–3, %).

We obtained a significant P value of the exploratory test of beta diversity between BD patients and normal individuals. The bacterial taxa of BD patients and those of normal individuals had a significant distance, suggesting that the compositional alteration is obvious between BD patients and normal individuals.

## Discussion

We have previously reported that Th17 cell frequencies increased significantly and they showed excessive responses against several proinflammatory cytokines, such as IL1, IL23 and TNFα in patients with BD [8, 9].

Our hypothesis is that some type of dysbiosis may have an association with the skewed Th cell activation in patients with BD.

We thus conducted a metagenomic analysis of fecal samples in patients with BD. We observed that the phylum *Actinobacteria*, especially the order *Bifidobacteriales*, increased significantly and the phylum *Firmicutes*, especially the order *Clostridiales*, decreased in BD patients (Fig 2).

The bacterial compositional changes of BD patients shown here may represent one type of BD intestinal dysbiosis.

Recent studies suggested that gut microbiota affected development and maintenance of systemic immunological function [38]. The compositional alterations were suggested to associate with the skewed immune responses and emergence of self-reactive Th cells [38].

Animal studies using germ free mice reported that some bacterial species separately promoted arthritis through activation of Th17 cells [16, 17]. Indeed, oral intake of *Lactobacillus bifidus* rapidly induced arthritis in genetically modified germ free mice [16].

In concordance with the inductive effect of *Lactobacillus* on the systemic inflammation, we found that *Lactobacillus* species had relatively large effect sizes in BD microbiota (Fig 2).

*Clostridium* species were suggested to activate regulatory type T cells and then modulate mucosal immune system through the production of short chain fatty acids [39].

In the current study, at the class level, *Actinobacteria*, *Coriobacteria* and *Bacilli* had large effect sizes in BD patients, whereas *Clostridia* had a large effect size in normal individuals (Fig 2). These classes include relatively abundant commensal bacteria in humans and the physiological aspects were well-studied using metagenomic analyses and laboratory assays [40].

The species composition of the intestinal microbiota is suggested to be determined by two major mechanisms of gene function, namely the bacterial capabilities for utilizing dietary substrates and their tolerance to gut environment, such as pH and bile salt concentrations [40]. *Bifidobacterium* and *Lactobacillus* are major lactate producing and pH regulating bacteria with the consumption of hexose sugars [40]. *Coriobacterium* species are also lactate producing bacteria [40].

In contrast to the lactate production, several genera of the order *Clostridiales*, including *Veillonella* species, are able to utilize lactate and produce butyrate or propionate [41, 42].

Compositional changes from short chain fatty acid producing bacteria to lactate producing bacteria may have an association with the pathophysiology of BD through the lactate overproduction, which are generally considered to be detrimental for intestinal homeostasis [43].

Recently, a microbe identification microarray analysis of BD saliva was conducted in combination with a culture assay of saliva and mucosa [44]. In the array analysis, *Bifidobacterium dentium* and *Prevotella histicola* were more prevalent in BD saliva compared with those of normal individuals.

*Campylobacter concisus* and *Clostridiales* species colonized less frequently in BD saliva. *Neisseria* and *Veillonella* were frequently isolated from mucosa of normal individuals. Similar compositional changes of microbiota were found in cultivable bacteria from periodontal sites [45] and pustular skin lesions [46] in patients with BD.

A cutting-edge analysis of BD fecal metagenomics done by Consolandi unequivocally showed a characteristic bacterial compositional changes, which were similar to our current results to some extent [47]. Importantly, it was revealed that butyrate production of gut microbiota decreased significantly in BD patients compared with that in normal individuals. The result is consistent with our hypothesis that dysregulated short chain fatty acid production of gut microbiota may occur in BD patients.

Consolandi et al. reported decreased alpha diversity of gut microbiota in BD samples [47]. Here, we observed comparable alpha diversity between BD patients and normal individuals. It is possible that BD disease activity of patients participating in our study may be too mild at the time of sample collection to lead to alpha diversity loss. These results highlight the need for further investigations of the diversity in BD patients.

It is conceivable that medications used for treatment of BD patients have various effects on the intestinal microbes. Nonetheless, it was reported that colchicine used here (daily doses of 0.79 ± 0.26 mg) hardly affected the gut microbes and host intestinal mucosa [25–27]. Our preliminary study suggested that cyclosporine (daily doses of 125 and 50 mg) and azathioprine (a daily dose of 75 mg) had marginal effects on the gut microbes (manuscript in preparation).

It was reported that prevalent microorganisms of saliva samples were detectable in the fecal samples in metagenomic and gene expression analyses [48]. Simultaneous assessment of oral and gut microbiota may allow us to explore the distinctive effects of them on the pathophysiology of BD.

In conclusion, we observed a characteristic compositional change of gut microbes in patients with BD. *Actinobacteria* and *Lactobacillus* species were more prevalent and *Clostridia* was less frequent in gut microbiota of BD patients than in those of normal individuals. Further studies are needed to elucidate how the dysbiosis contributes to the immune dysregulation in patients with BD.
